# Chinese University English Teachers’ Professional Learning through Academic Reading on Social Media—A Mixed-Methods Approach

**DOI:** 10.3390/bs12100390

**Published:** 2022-10-12

**Authors:** Suhe Ji, Xiaoqing Qin, Ke Li

**Affiliations:** 1 School of Foreign Languages, Central China Normal University, Wuhan 430079, China; 2 School of Journalism and Communication, Wuhan University, Wuhan 430072, China; 3 Center for Studies of Media Development, Wuhan University, Wuhan 430072, China

**Keywords:** Chinese university English teachers, academic reading, professional development, social media, content knowledge, pedagogical content knowledge

## Abstract

Teachers’ professional learning on social media has received growing attention recently, but research into teachers’ academic engagement on social media remains limited. This study aimed to examine what factors motivated university English teachers to engage with academic reading on social media. The determinants of academic reading on social media were identified by semi-structured interviews, which were further validated by quantitative investigation. The results showed that participants’ perception of values of content knowledge, pedagogical content knowledge, and students’ participation in the classroom affected their intentions toward commitment to academic reading on social media. Additionally, the results revealed that perceived value of pedagogical content knowledge mediated the relationship between perceived value of content knowledge and participants’ intentions toward academic reading on social media while also mediating the relationship between perceived value of students’ participation in the classroom and the participants’ intentions toward academic reading on social media. These findings yield implications for the professional development of university teachers and the development of pedagogical content knowledge.

## 1. Introduction

Technological advancements have made social media increasingly pervasive. Typical social media features (such as shares, comments, and linking to journal articles) facilitate connections and interconnections, which in turn promote access to information related to professional learning over time. Research on social media in scholarship suggests that social media has gradually become a platform for teachers’ (including K-12 teachers, university teachers, and professors in higher education) professional development, communication, and self-directed learning [1] and that, on such a platform, academic openness has blurred the boundary between the literature and scholars’ engagement [2]. The mobility and autonomy of self-directed learning on social media contribute to wide and deep engagement. It was reported that 55% of teachers used social media for professional development, and teachers’ use of social media for professional development purposes has grown twice as fast as it has for personal use since 2013 [3]. In addition, teachers’ autonomous learning on social media has become a peer-to-peer approach to constructing subject-directed knowledge that is different from the traditional top-down course-based professional development model for teachers [4].

Teachers’ professional development arises in both formal and informal contexts. However, to a great extent, 90% of teachers’ new knowledge is acquired through informal learning [5]. Social media, such as Facebook [6], WeChat [7], and Twitter [1,8], has promoted a culture of collaboration in the informal learning environment and led to new insights, thus facilitating the combination of teachers’ research and teaching itself. This integration of theory and practice can foster academic reading on social media, which becomes a self-constructed approach to professional development in informal learning contexts. However, due to the differences in culture, policy, and practice of different countries, it is necessary to isolate a specific context in order to study teachers’ professional learning [9], including research into academic engagement on social media. Therefore, the current study focused on teachers’ academic engagement on social media in the context of mainland China because social media has integrated into the daily lives of university teachers worldwide [10,11], and because using social media for academic purposes has also become pervasive in Chinese university teachers’ lives. In China, university teachers’ professional learning on social media is associated with academic disciplines. For example, a study by Zhao and Zong showed that psychological and educational journal articles were the two most frequently read types of papers on WeChat [12], which suggests humanities scholars read more journal articles on social media than scholars of other disciplines. Among these humanities scholars, Chinese university English teachers are a group of individuals with especially distinct characteristics. For one reason, Chinese university English teachers are considered as the largest English teaching group, owing to the largest number of students learning English as a second language in the world. Additionally, the majority of Chinese university English teachers are active social media users for field-specific and research-oriented purposes. This is because they have increasingly adapted to using social media for information searching and academic reading with the rich and authentic language learning materials found on social media [7]. They also have fewer language barriers preventing them from adopting social media (e.g., Facebook, Twitter) in research and teaching practices. However, some Chinese university English teachers may heavily rely on the academic resources on social media because not all of them can easily access English journals published outside of China [13]. Meanwhile, a large number of Chinese university English teachers found themselves struggling to fulfill the academic productivity demands made by policy makers in the “publish-or-perish” context [13,14,15]. Under this heavy pressure to publish journal articles in international journals, they are expected to invest more time and effort engaging with academic reading outside of their routine teaching. Against this background, we seek to explore what motivates Chinese university English teachers’ commitment to academic reading on social media, which may be useful for tapping into the mechanisms involved in university teachers’ professional development as supported by new technologies in the informal learning context.

## 2. Literature Review

### 2.1. Toward Defining Academic Reading on Social Media

Different from leisure reading and pleasure reading, academic reading is considered as an activity conducted by teachers and/or researchers to quickly access a large amount of academic information [16]. In the field of second/foreign language teaching, academic reading involves language teachers’ motivation to read research articles, access to academic resources, and the time required to engage with it outside the formal learning context [9]. Previous research has found that language teachers’ academic reading significantly and deeply influenced subsequent classroom teaching [17], and teachers’ critical reading of research articles has been proven to be beneficial to their professional development [18]. It has been claimed that interactions within academia have enabled language teachers, especially novice teachers, to build connections with professionals in a shared academic community [19]. 

However, the conceptual definition of academic reading on social media has not yet been defined. Some researchers consider academic reading on social media (e.g., reading more articles, books, and other publications related to teaching and research on social media [20]) as university scholars using social media in order to access academic resources and read academic information for work-related purposes [11,20]. This description briefly depicts the most important media characteristics of academic reading on social media. Nevertheless, this study claimed that academic reading on social media was not only a change in the media of academic materials, from traditional journals to digital sharing articles on social media [21], but also a change in the reading patterns of scholars, from academic library reading to a pattern of academic reading embracing information expansion and interaction [22]. These noticeable changes highlighted differences between traditional academic reading (e.g., searching and retrieving academic information from libraries and/or online libraries [20]) and academic reading on social media. First, reading information on social media is obtained through official accounts pushing articles [12,23] or through sharing [24], which suggests that access to academic reading materials is not completely dependent on active searching but can be facilitated by official accounts. Although academic reading on social media was critical reading to a great extent, it was different from traditional academic reading in terms of seriousness [25]. Second, regarding the context of academic reading on social media, it can take place on the subway, in the corridor, while traveling, during a short break, etc. In this sense, academic reading on social media was a form of self-directed informal learning [26,27]. Third, academic reading on social media had a greater advantage in relation to interactions in academic research communities than traditional academic reading because of the role of social media in sharing, commenting, accessing, and recommending [24,28]. If scholars follow the same official account and read the same academic articles, an invisible academic community may be formed [6]. These differences could lead to a growing number of individuals switching from traditional academic reading to academic reading on social media, resulting in great changes in reading patterns among scholars. This may also represent a new trend regarding teachers’ professional learning in the informal learning context.

### 2.2. Analytical Framework

#### 2.2.1. Technology Acceptance Model and Related Models

As mentioned above, defining academic reading on social media from the perspective of media technology focuses on its technical behavior. Accordingly, the explanation of the influencing factors may be based on the acceptance behavior of a specific technological system. Therefore, the technology acceptance model (TAM) has been widely used to explain teachers’ acceptance of certain technology use [29,30]. 

The prevalence of the TAM being used to explain how and why technologies are adopted by certain groups has been greatly acknowledged. The TAM proposes that perceived usefulness and perceived ease of use are the top factors that influence the attitudes and intentions of individuals toward adopting or rejecting technology use [31], and the model has been utilized and validated in research on social media in the higher education context [32,33]. However, these determinants of behavioral intention were associated with the qualities of the system, information, and user satisfaction [34], ignoring environmental or economic factors [35] and neglecting the credibility and security of the information on social media [36]. Taking the TAM as the underpinning model, the Unified Theory of Acceptance and Use of Technology (UTAUT) model integrated other theories and models [37], further improving the explanation of user acceptance and adoption of technology usage (e.g., social media), as claimed by the study of Venkatesh et al. that reported that the UTAUT model could explain 70% of the variance in behavioral intention [37]. The UTAUT model suggests that there are three direct determinants of the intention to use technology, including performance expectancy, effort expectancy, and social influence [37]. Based on the UTAUT model, it was found that performance expectancy and social influence positively affected science and technology scholars’ intention to use social media for academic purposes; meanwhile, effort expectancy and social influence negatively affected science and technology scholars’ intention to use social media [38]. However, a study by Dermentzi and Papagiannidis showed that performance expectancy, facilitating conditions, and users’ habits affected the behavioral intention to engage with academia [39]. These findings indicate that the application of the UTAUT model in detecting the determinants of intentions to use social media for academic engagement highlights the important characteristic of the user friendliness of a system, and this technology-based theory may be more appropriate for explaining certain media behaviors. However, regarding the inquiry into university teachers’ beliefs and their motivation to committing to professional development, the theoretical framework of teachers’ knowledge base has been applied to explore how teachers’ perceived value of knowledge influenced their intentional behaviors [40]. Therefore, in the present study, we used a teachers’ knowledge base framework to explain university teachers’ intentions toward academic reading on social media.

#### 2.2.2. Teachers’ Knowledge Base Framework

Shulman’s theoretical framework of teachers’ professional knowledge enabled us to categorize teachers’ professional knowledge, and helped identify the intentions of teachers regarding engagement with academic reading on social media for professional learning [41]. The framework of teachers’ professional knowledge maintains that the conception of teacher knowledge includes content knowledge, general pedagogical knowledge, and pedagogical content knowledge, as well as knowledge of general pedagogy, curriculum, learners, educational contexts, and educational purposes [41]. Previous research into teachers’ professional knowledge has assumed that content knowledge and pedagogical content knowledge were at the heart of teachers’ competence [42,43,44]. In terms of the relationship between content knowledge and pedagogical content knowledge, there was consensus that content knowledge (the amount of teachers’ knowledge [41]) was a necessary prerequisite for pedagogical content knowledge (the transformation of content knowledge for the purpose of teaching [45]) [46,47]. In addition, empirical studies have confirmed that content knowledge and pedagogical content knowledge positively and significantly affected teachers’ teaching practices and students’ achievements [46,48,49].

However, the issue of teachers’ professional knowledge is not confined to external knowledge per se. The perceived value of professional knowledge was positively related to teachers’ beliefs [50], and this belief is particularly related to the intention to engage with professional development programs. A study by Hwang et al. revealed that perceived value of content knowledge and perceived value of pedagogical content knowledge significantly influenced teachers’ intentions to participate in professional development activities (e.g., in-service learning programs, keynote lectures, and self-study activities) [40]. This finding suggests that teachers can consciously perceive the value of certain behaviors to their professional development and teaching practices, and then willingly commit to the engagement; in other words, perceived value of content knowledge and pedagogical content knowledge significantly affect teachers’ engagement with professional learning outside the formal learning context. Academic reading on social media is a spontaneous and conscious learning behavior. Although it was fragmented and informal, it was still driven by teachers’ beliefs and intentions [51,52,53]. As such, this study assumed that it was feasible to explain teachers’ intentions to engage with academic reading on social media based on the conceptual framework of Shulman’s knowledge base framework. Under this framework, we noted that both perceived value of content knowledge and perceived value of pedagogical content knowledge may affect teachers’ willingness to conduct academic reading on social media. However, due to the lack of relevant empirical research, it cannot be ruled out that other influencing factors may have an impact on academic reading on social media. Therefore, we opened up the following research question to guide this study: 

What factors and structures influence Chinese university English teachers’ engagement with academic reading on social media?

## 3. Research Design

In line with the research question, we employed an exploratory sequential design [54], which was a two-phase mixed-methods approach. The first phase was qualitative research based on semi-structured interviews that was followed by the second phase, quantitative research that employed structural equation modeling (SEM) for statistical analysis. Because there were few existing studies on academic reading on social media, and even less research tapping into factors that influenced teachers’ engagement with academic reading on social media, an initial explorative interview study should be conducted to probe into potential factors that can influence academic reading on social media. Based on Shulman’s knowledge base framework, an interview outline and a priori themes were formed before conducting interviews. Hypotheses were then formulated in the quantitative research part of the study, based on the results of the former interview study. These hypotheses were further tested with large-scale testing using SEM.

## 4. Qualitative Research

### 4.1. Interview Questions

According to the above literature, it was found that the factors that may affect teachers’ engagement with academic reading on social media included perceived value of content knowledge and perceived value of pedagogical content knowledge [6,40,41]. Thus, based on a priori themes identified by previous research, interview questions were informed that were set in open-ended prompts. Interview questions included the following:

(1) Have you followed some official accounts related to teaching or academic research on social media? Do you often read academic information on social media?
(2) Can you explain what aspects of academic reading on social media you find most valuable? Why?
(3) Will you use resources and information obtained from academic reading on social media in classroom teaching? Why and how do you use these resources?
(4) What are the effects of the application of the resources obtained from academic reading on social media on classroom teaching?

### 4.2. Participants and Interviews Procedures

Based on purposive sampling, a total of 12 Chinese English teachers were recruited from three comprehensive universities in a provincial city in central China. In line with the principle of maximum differentiation, this study took into account the differences in gender, years of teaching experience, and education level of the respondents when selecting the participants, and the study required that the participants should have some academic reading experience on social media. The interviews were conducted in the form of one-on-one sessions. Each interview lasted about 30 min to one hour, which was recorded after the consent of the participant was obtained. The recorded materials were then transcribed and coded. After analyzing and coding the interview content of the 12 interviewees, it was found that no new theme could be generated. According to the suggestions of Francis et al. regarding data saturation [55], three more interviews were conducted. Data saturation was finally achieved after interviews with a total of 15 respondents. Among them, three were male and twelve were female. In terms of years of teaching experience, the longest length of teaching experience was 39 years, and the shortest length was 3 years. There was one participant with a bachelor’s degree, 11 participants with a master’s degree, and three with a doctorate degree.

### 4.3. Results of the Interviews

With regard to the behavior of academic reading on social media, the interview aimed to explore how often and for what reason Chinese university English teachers adopted social media for professional learning purposes. It was found that participants tended to use more than one type of social media, for example, they preferred to combine a certain type of social media with one or more other types. WeChat was the most popular type of social media. Two-thirds of the participants reported that the primary reason for using WeChat for academic reading was due to the greater availability of journals and journal articles, which expanded their reading scope via official accounts pushing content. The results also found that participants used several types of social media (such as Weibo, Facebook, and Twitter) for searching scholarly material (such as video lectures) and authentic English learning material for students. Regarding the frequency of using social media for academic purposes, all the participants reported that they read academic information on social media every day for at least half an hour, which indicates that academic reading on social media is important. Meanwhile, the results revealed that academic reading on social media was not all scattered and informal. Some participants reported that they would arrange a specific time for academic reading on social media and make some notes, classify information, and search for related academic articles. This finding suggests that the penetration of social media into academic work is deeper than expected. 

In terms of the determinants of participants’ intention to read academic material on social media, based on Hesse-Biber’s coding method [56], a top-down template analysis was adopted to analyze the transcribed texts. This analytical approach enabled us to introduce a priori themes identified by previous research. We used these a priori themes to guide constant comparative revision of the coding transcripts. Meanwhile, when there was no suitable a priori theme that was associated with segments of text, a new theme was generated. Therefore, this coding process involved both a top-down and a bottom-up approach. The bottom-up approach involved creating new themes based on the grounded reading of the texts, which included three major steps [56,57]: (1) independently reading each transcript thoroughly, (2) identifying new patterns, and (3) generating new themes based on the coded patterns. Through the template analysis process, three themes were identified. The two a priori themes that were detected from previous research were perceived value of content knowledge and perceived value of pedagogical content knowledge. The newly created theme was perceived value of students’ participation in the classroom. 

Perceived value of content knowledge refers to teachers’ targeted academic reading on social media based on their own professional development needs. The main finding revealed that participants were aware of the value of academic reading on social media to their subject-matter knowledge and professional learning. It suggests that teachers’ professional knowledge is largely accumulated through an amount of self-initiated academic reading. As Participant 3 said about her reading experience on social media:

*Some articles on public accounts have greatly inspired my understanding on language acquisition theories. It can deepen my understanding of knowledge that used to be difficult to grasp.*

Perceived value of pedagogical content knowledge refers to the value judgment regarding whether teachers’ knowledge and teaching strategies can be effectively integrated. The interviews showed that participants valued the accessible learning resources on social media and intended to implement these resources in their classroom teaching, mainly attributed to rich academic resources on social media offering alternative ways to expand, organize, or modify their teaching for a particular topic. Participant 12, an English language teacher teaching linguistics, perceived that academic reading on social media was useful to her teaching practices:

*I watch short videos on social media. The short videos of famous linguists’ lectures on linguistics are very helpful to my linguistics teaching. I play these video lectures in class; it can expand students’ learning beyond the textbook.*

Perceived value of students’ participation in the classroom refers to teachers’ assessment of the value of resources on social media to students’ active classroom participation. Almost half of the participants reported that they implemented new ideas learned from academic reading on social media when they detected students’ confusion, distraction, or misunderstanding. The result indicates that valuable information gathered from social media can maintain students’ interest, grasp students’ attention, and facilitate classroom learning. Participant 9, a college English teacher, taught a large class packed with more than 100 students; she felt enormous benefits from academic reading on social media. She said:

*My college English class is so big and crowded with boys. It is hard for me to hold their attention. But sometimes I use some resources from social media that I think are interesting to my students. It worked! I find that social media has so many curricular resources and amazing connections. I will continue to use these helpful resources in my English class.*

The above results showed that teachers’ academic reading on social media was influenced by the perceived values of content knowledge, pedagogical content knowledge, and students’ participation in the classroom. These results concurred with Hwang et al., who proved that teachers’ perceived value of content knowledge and perceived value of pedagogical content knowledge affected their intent to participate in professional development programs [40]. Moreover, this finding was in line with the concept of utility value that was considered as the subjective instrumental evaluation of behavior [58]. Accordingly, we put forward the following hypotheses:

**H1:**  *Perceived value of content knowledge positively influences Chinese university English teachers’ intentions toward engagement with academic reading on social media.*

**H2:**  *Perceived value of pedagogical content knowledge positively influences Chinese university English teachers’ intentions toward engagement with academic reading on social media.*

**H3:**  *Perceived value of students’ participation in the classroom positively influences Chinese university English teachers’ intentions toward engagement with academic reading on social media.*

In addition, the results of interviews showed that there were relationships among the three determinants of intent to read academic material on social media. First, the results confirmed that content knowledge directly influenced the development of subject pedagogical knowledge [45,47]. The findings revealed that teachers’ capacity to make value judgments on pedagogical content knowledge development was grounded on their subject-matter knowledge. It suggests that teachers’ understanding of subject knowledge is a primary factor that influences their evaluation of the usefulness of this knowledge to teaching practices. Participant 11, a novice college English teacher, described in his interview how he perceived the information he read on social media:

*I know that I must be cautious when adopting information from social media. I should judge whether it is useful to my understanding of the text first, and whether it can be used to improve my teaching.*

Second, the interviews revealed that teachers’ perception of the students’ responses in classroom learning influenced their implementation of different teaching strategies, which indicates that teachers’ perceived value of students’ participation in the classroom has an impact on their perceived value of pedagogical content knowledge. As an example, Participant 5, who is teaching comprehensive English, described her experience with academic reading on social media:

*When I am preparing the lesson plan, I find that some parts of the text are difficult for the students to understand, then I know that I should make some changes or bring some new ideas from social media to make it easier for my students to follow the lesson.*

In this sense, based on previous research and the above findings, the following hypotheses were formed:

**H4:**  *Perceived value of content knowledge positively influences perceived value of pedagogical content knowledge.*

**H5:**  *Perceived value of students’ participation in the classroom positively influences perceived value of pedagogical content knowledge.*

Given that academic reading on social media was viewed as an intentional behavior, and that the evidence from the above interviews revealed three influencing factors and complex internal relationships among these factors, it may be hypothesized that perceived value of pedagogical content knowledge played a mediating role between intentional behavior and the other two influencing factors. Thus, we proposed the following hypotheses:

**H6:**  *Perceived value of pedagogical content knowledge mediates the relationship between perceived value of content knowledge and intentions toward academic reading on social media.*

**H7:**  *Perceived value of pedagogical content knowledge mediates the relationship between perceived value of students’ participation in the classroom and intentions toward academic reading on social media.*

**Figure 1:**
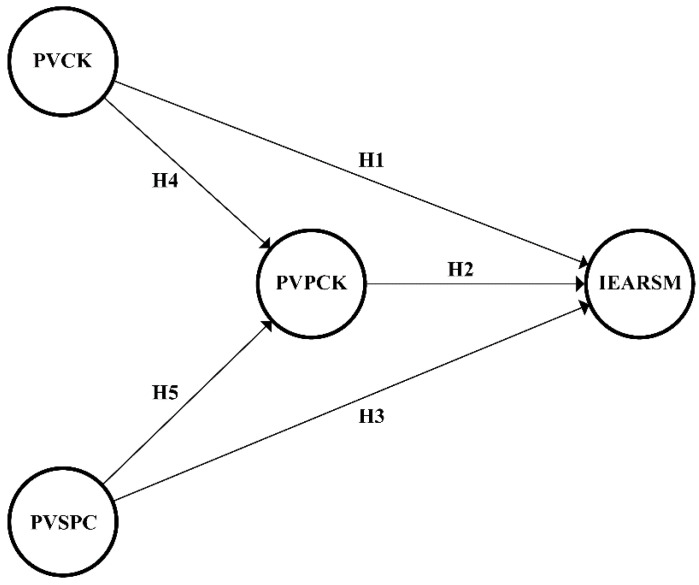
A proposed structural model with constructs. PVCK = perceived value of content knowledge, PVPCK = perceived value of pedagogical content knowledge, PVSPC = perceived value of students’ participation in the classroom, IEARSM = intentions toward engagement with academic reading on social media.

According to the hypotheses put forward by the first phase of the study, the qualitative research phase aimed to explore the interrelations between teachers’ perceived value of content knowledge, perceived value of pedagogical content knowledge, perceived value of students’ participation in the classroom, and intentions toward engaging with academic reading on social media. Accordingly, a structural model was proposed, which is shown in Figure 1.

## 5. Quantitative Research

### 5.1. Measurements

The online questionnaire employed to collect data was categorized into three sections. The first section asked for general information about the participants’ usage of social media for academic purposes. The second section explored participants’ intentions toward engaging with academic reading on social media, as well as participants’ perceptions of the values of content knowledge, pedagogical content knowledge, and students’ participation in the classroom. The third section covered the demographic information of the participants. The first section of the questionnaire intended to investigate Chinese university English teachers’ behaviors in relation to academic reading on social media, including the types of social media that they preferred for academic reading, the frequency of reading academic material on that social media, and the experience of academic reading on social media. This inquiry into the general information relating to academic reading behaviors on social media offered detailed background information about the real experience of academic reading on social media. In the second section, teachers’ engagement with academic reading on social media was measured by referring to Tenopir et al. [11,20]. Perceived value of content knowledge was measured by three items adopted from Baumert et al. [46], Hwang et al. [40], and statements from participants of the semi-structured interview. Perceived value of pedagogical content knowledge was assessed using the items revised from Hwang et al. [40] and Park and Oliver [45]. Perceived value of students’ participation in the classroom was measured using items generated from the interviews in the first phase of this study. In the third section, participants were asked to fill out their demographic information, such as gender, age, and years of teaching experience. Responses to items were measured by 7-point Likert scales, with 1 representing strongly disagree and 7 corresponding to strongly agree. The variables, items of measurements, and sources are detailed in Table 1. The main items of the questionnaire are available as Supplementary Material.

### 5.2. Data Collection

The target participants of the survey were active Chinese university English teachers who were expected to have experience in academic reading on social media. Thus, snowball sampling was used to conduct the survey. The online questionnaire was first distributed to 18 university English teachers, who were from the same province where the interviewees were recruited in the qualitative research phase. The questionnaire was then distributed to other university English teachers through invitations sent by the initial participants. Respondents participated in this investigation voluntarily and gave informed consent. If they did not have certain social media academic reading experience, they could withdraw from answering the questionnaire. As a result, a total of 289 respondents were invited, and a total of 157 participants finished the questionnaire. Excluding some questionnaires with incomplete information, 132 valid questionnaires remained. Among these included participants, nine were males (7%) and 123 were females (93%). With regard to the age of the participants, 13 (10%), 84 (64%), 25 (19%), 9 (7%), and 1 (1%) were 20–30, 31–40, 41–50, 51–60, and above 60 years of age, respectively. In terms of the years of teaching experience, 50 (38%) of the participants had less than 10 years of teaching experience, 62 (47%) of the participants had 11–20 years of teaching experience, and 20 (15%) of the participants had more than 20 years of teaching experience (see Table 2).

### 5.3. Data Analysis

SmartPLS 3.3.2 was used to analyze the data, which is a partial least squares structural equation modeling (PLS-SEM) analysis and processing software based on the PLS algorithm. Compared to covariance based structural equation modeling (CB-SEM), PLS-SEM is more suitable for the data analysis of small samples and has no mandatory requirements for data distribution. Therefore, PLS-SEM was more suitable for the data analysis in this study. The sample of 132 questionnaires met the sample requirements of PLS-SEM [59]. 

Data analysis involved the following steps: The first step was to detect the issue of common method bias. Common method bias was caused by the measurement method rather than the causal relationships in the model being studied [60]. It can lead to validity bias in measurements. Therefore, the primary step of the data analysis was to detect whether the model was contaminated with unexpected bias. The second step was to assess the measurement models. Evaluation of the reflective measurement models was undertaken in order to test the reliability and validity of the measurement models to ensure each construct was accurately measured. During this process, the main indicators and their measurements involved: (1) indicator reliability, which was measured by factor loadings; (2) internal consistency, which was assessed by Cronbach’s α and composite reliability (CR); (3) convergent validity, which was judged by the value of average variance extracted (AVE); and (4) discriminant validity, which was examined by heterotrait–monotrait ratio (HTMT). The third step was to assess the structural model by examining the collinearity issues, path coefficients, coefficient of determination (R^2^), effect size (f^2^), predictive correlation (Q^2^), and standardized root mean square residual (SRMR). The purpose of the assessment of the structural model was to evaluate whether the model met the requirements and verified the hypotheses. The last step was to examine the mediation effects. Specifically, it was necessary to further examine the mediating role of perceived value of pedagogical content knowledge in the model.

### 5.4. Results

#### 5.4.1. Common Method Bias

According to the guideline suggested by Kock [60], a full collinearity test result lower than 3.3 has been proposed as an indication of no common method bias being present. In this study, the full collinearity test results of the four constructs were lower than 3.3, indicating that they were absent of the issue of common method bias.

#### 5.4.2. Assessment of Measurement Models

For the assessment of measurement models, based on the suggestion proposed by Hair et al. [59], factor loading, Cronbach’s α, and CR should be greater than the threshold value of 0.7; the cut-off value of AVE should be greater than 0.5; HTMT should be less than 0.9; and the results of the HTMT ratio in the lower and upper bounds of 95% should be absent of the value of 1 after being computed through bootstrapping. From the results shown in Table 3, all indicators met the requirements, indicating that the measurement models have good reliability and validity. Table 4 reveals that the HTMT criterion has good discriminant validity.

#### 5.4.3. Assessment of the Structural Model

**Figure 2:**
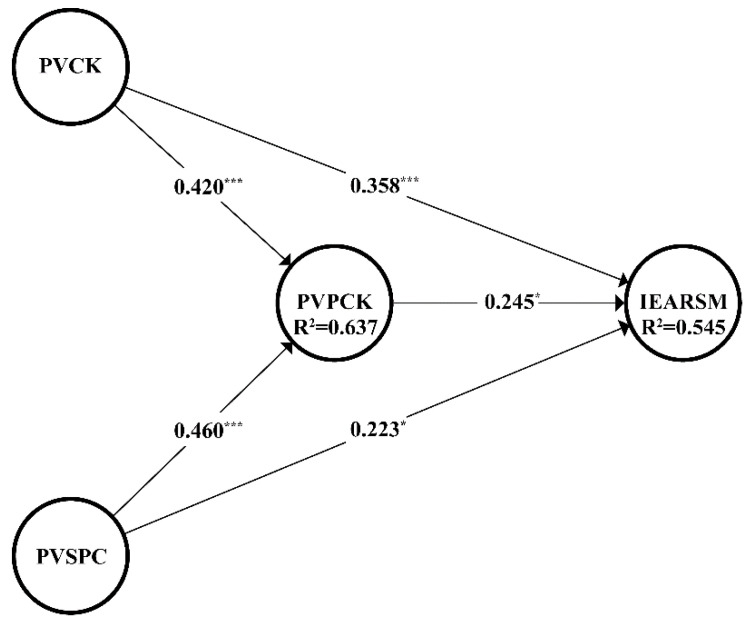
The structural model with main results. Notes. * *p* < 0.05, *** *p* < 0.001. PVCK = perceived value of content knowledge, PVPCK = perceived value of pedagogical content knowledge, PVSPC = perceived value of students’ participation in the classroom, IEARSM = intentions toward engagement with academic reading on social media.

As shown in Table 5, the results of the variance inflation factor (VIF) values were lower than the cut-off value of 5 proposed by Hair et al. [59], suggesting the absence of a collinearity problem. As the representations of the hypothesized relationship between constructs, the path coefficients were calculated by bootstrapping to obtain the t value. It is suggested that a t value greater than 1.96 denotes a 0.05 significance level, greater than 2.58 indicates a 0.01 significant level, and greater than 3.29 indicates a 0.001 significance level [61]. The results shown in Table 5 and Figure 2 revealed that all the five directional hypotheses were empirically supported. The coefficient of determination represents the strength of the model’s predictive power. In PLS-SEM, R^2^ values of 0.75 represent substantial predictive power, R^2^ values of 0.50 are described as moderate predictive power, and a value of 0.25 represents weak predictive power [59]. According to the R^2^ values shown in Table 5, the structural model had moderate predictive power for teachers’ engagement with academic reading on social media (R^2^ = 0.545), and it had moderate power for perceived value of pedagogical content knowledge (R^2^ = 0.637). The prediction correlation f^2^ represents the accuracy of prediction between constructs. Guidelines for evaluating f^2^ show that an effect size value of 0.02 indicates a small effect, an effect size value of 0.15 a medium effect, and 0.35 a large effect [59]. In this study, the results show that perceived value of content knowledge, perceived value of pedagogical content knowledge, and perceived value of students’ participation in the classroom had small effects on teachers’ intentions toward academic reading on social media (f^2^_PVCK≥IEARSM_ = 0.128, f^2^_PVPCK≥IEARSM_ = 0.048, f^2^_PVSPC≥IEARSM_ = 0.048). Meanwhile, perceived value of content knowledge and perceived value of students’ participation in the classroom had medium effects on perceived value of pedagogical content knowledge (f^2^_PVCK≥PVPCK_ = 0.286, f^2^_PVSPC≥PVPCK_ = 0.342) (see Table 5). Moreover, the Q^2^ values were greater than 0, suggesting that the model has good predictive power. The model fit index in PLS-SEM was represented by SRMR. If the value of the estimated SRMR of the model is less than 0.08, it suggests that the model fits well [62]. The SRMR value of the model in this study was 0.074, indicating a good model fit.

#### 5.4.4. Assessment of Mediation Effects

The assessment of mediation effects involved two aspects. First, according to Zhao et al.’s approach to identifying mediation effect [63], we examined the mediating role of perceived value of pedagogical content knowledge in the model, and found that it played a complementary mediation role in both mediation paths. Second, to further describe the strength of the mediation effects, the variance accounted for (VAF) was calculated with reference to the method proposed by Hair et al. [61]. As shown in Table 6, VAF for perceived value of pedagogical content knowledge in the two mediation paths was 22.34% and 33.43% respectively, which verified that hypotheses H6 and H7 were both supported. 

## 6. Discussion

In this study, we explored Chinese college English teachers’ motivations and intentions toward devoting themselves to professional development in an informal learning context. Specifically, we have focused on what drove teachers toward engaging in academic reading on social media in their fragmented time. The results of this study found that teachers’ perception of the values of subject-matter knowledge, pedagogical knowledge, and learners influenced their intention and willingness to commit to academic reading. The result was consistent with the proposition that teachers’ beliefs about learning, teaching, and learners influenced their perception of new information, and thereby guided their subsequent behaviors [64]. Beliefs served as a filter for information screening [65] and acted as motivation that enabled teachers to be imbued with emotive engagement. 

The results of the interviews confirmed the findings of Hwang et al., in that teachers’ perceived value of subject-matter knowledge and perceived value of pedagogical content knowledge influenced teachers’ intentions and attitudes toward engaging in professional development activities [40]. This finding reveals teachers’ motivations regarding maintaining or developing their professional competence through ongoing self-initiated learning and toward integrating professional development into teaching practices. To a large extent, this motivation was extrinsic motivation, driven by the instrumental view of the value of engagement with academic reading on social media for positive effects on teaching practices [9]. As Borg and Liu [9] concluded, Chinese university English teachers seeking academic reading from various publications may largely be attributed to the need to solve local teaching problems. In addition, the interviews found that Chinese university English teachers’ perceived value of content knowledge influenced perceived value of pedagogical content knowledge, which concurred with previous research suggesting that teachers’ beliefs about subject-matter knowledge influenced their beliefs about students’ learning and development [66]. This result echoed the empirical finding that teachers’ subject-matter knowledge was a prerequisite for the development of pedagogical knowledge [45,47]. The empirical research has proven that content knowledge leads to the development of pedagogical content knowledge [47], and deficits in content knowledge limit the potential scope of developments in pedagogical content knowledge [46]. As Shulman claimed [41], teachers’ broad and deep understanding of content knowledge was the primary stage of imparting explanations of knowledge; in other words, pedagogical content knowledge was based on the particular form of teachable content knowledge [67]. Moreover, another main finding of the interviews was that teachers’ evaluation of the value of resources to students’ participation in the classroom emerged as a new factor that influenced teachers’ adoption of instructional strategies and accordingly guided their purposeful searching for academic reading on social media. It indicates that teachers’ assessment of the characteristics of material on social media is largely based on their understanding of students’ abilities, interests, and prior knowledge; teachers’ evaluation of the adaption of this material into classroom teaching is dependent on their prediction of students’ reactions and feedback. This result aligned with the study by Park and Oliver who believed that teachers should integrate all their understandings of materials, students, and subject-matter knowledge and apply them to teaching practice by appropriate responses to students [45]. 

The results of the structural equation model showed that the structural model explained 54.5% of variance (R^2^ = 0.545) in teachers’ engagement with academic reading on social media as defined by perceived values of content knowledge, pedagogical content knowledge, and students’ participation in the classroom. In other words, perceived values of content knowledge, pedagogical content knowledge, and students’ participation in the classroom were identified as the determinants of teachers’ intentions toward engagement with academic reading on social media. In addition, the effect size results showed that perceived value of content knowledge had an almost medium effect on teachers’ intentions toward academic reading on social media (f^2^_PVCK__≥IEARSM_ = 0.128). It indicates that teachers’ perceived value of content knowledge is a strong predictor of their endeavor to read academic materials on social media; thereby suggesting that the driving force of university English teachers’ engagement with academic reading on social media generally stems from the impetus to develop and attain professional competence. This statistically significant finding was in line with the proposition made by Borg that promoting teachers’ knowledge may facilitate their academic research engagement [68], as lack of knowledge was one of the most commonly cited reasons for Chinese university English teachers’ low engagement with academic research [9]; teachers therefore felt in dire need of professional research competence development [69]. Additionally, the findings of the SEM analysis revealed that teachers’ perceived values of content knowledge and students’ participation in the classroom had significant and positive effects on their perception of pedagogical knowledge (f^2^_PVCK≥PVPCK_ = 0.286, f^2^_PVSPC≥PVPCK_ = 0.342). These findings were in unison with the research of Park and Oliver [45] and Kleickmann et al. [47] in that teachers’ scholarly knowledge, understanding of the material organization, and detection of students’ responses had a determinate role in construction and development of teachers’ pedagogical content knowledge. And the finding also echoed the result of a case study of Rankin and Becker that teachers’ expectations of classroom culture affected their implementation of teachers’ knowledge [17]. 

In terms of the structure of the proposed framework, the SEM results did provide empirical evidence for the mediating role of perceived value of pedagogical content knowledge on the relationship between perceived values of content knowledge and teachers’ endeavor to read academic materials on social media, as well as on the relationship between perceived value of students’ participation in the classroom and teachers’ intentions toward engagement with academic reading on social media. Notably, the results shed light on the importance of the role of perceived value of pedagogical content knowledge, suggesting that teachers’ deeper levels of engagement with academic reading on social media may occur as a benefit of increases in perceived value of content knowledge and perceived value of students’ participation in the classroom, through increases in perceived value of pedagogical content knowledge. This finding was congruent with the findings of the study by Han et al., in that pedagogical content knowledge was closely associated with informal learning activities and that content knowledge was an important determiner of pedagogical content knowledge [70]. 

In sum, this study has theoretically contributed to the causal relationship in the teachers’ knowledge base framework and drawn attention to the significance of the mediating role of pedagogical content knowledge. Several studies have explored the relationship between content knowledge and pedagogical content knowledge [40,46,47]; however, few studies have probed into potential factors that influence perceived value of pedagogical content knowledge. The current study has advanced the conceptual understanding of the relationship between students’ feedback, reactions, and responses and teachers’ content knowledge and pedagogical content knowledge. Furthermore, the study has developed empirical evidence that perceived value of content knowledge had significant direct and indirect positive effects on teachers’ intentions toward engagement with academic reading. Therefore, this study has added to the current literature on university teachers’ motivations and intentions toward commitments to professional development in informal learning contexts. However, caution is required when generalizing the findings of this study and applying this model in other contexts as there are significant differences in frequency and preference regarding academic reading on social media for different teachers from various disciplines and countries [12,20,24,71]. Therefore, whether the conclusions of this study can be extended to other contexts remains to be verified, which is an important follow-up research issue. In addition, years of teaching experience, digital skills in social media usage, the content of academic reading on social media, reading contexts, and other factors may influence teachers’ academic reading on social media, which can be explored in subsequent studies. These factors may also help to improve the model of this study.

## 7. Conclusions and Limitations

With the aim of contributing to research on teachers’ commitment to academic reading under informal learning contexts in order to enhance professional learning, we explored factors and mechanisms that affected Chinese university teachers’ intentions toward engagement with academic reading on social media through a mixed-methods approach. In the qualitative research phase, we adopted the template analysis coding method to analyze the qualitative data through the two-level coding procedure. The results of the semi-structured interviews showed that three factors emerged as the determining factors of teachers’ intentions toward engagement with academic reading on social media: perceived value of content knowledge, perceived value of pedagogical content knowledge, and perceived value of students’ participation in the classroom. The results of the interviews also showed that teachers’ perceived values of content knowledge and students’ participation in the classroom influenced their perceived value of pedagogical content knowledge. Subsequently, the following quantitative research has verified the results of the interviews with a larger sample. The SEM results revealed that all the research hypotheses in this study were supported, and that the influence paths between variables showed that perceived value of content knowledge and perceived value of students’ participation in the classroom influenced teachers’ intentions toward engagement with academic reading on social media through the mediation effect of the perceived value of pedagogical content knowledge. This empirical finding indicates that Chinese university English teachers’ academic reading on social media shows the characteristics of autonomous learning in informal learning contexts. These findings are a concrete description of language teachers’ academic reading on social media and provide an empirical basis for the application of teachers’ knowledge base theory to explain the phenomenon of academic reading on social media. 

These findings may contribute to research into the academic research engagement of teachers. In our Chinese sample, most teachers had strong beliefs about themselves as teachers and as teacher educators for their important role in facilitating and enhancing students’ development [69]. This teaching-focused professional identification may foster their academic reading motivation for the application of academic knowledge to immediate and direct classroom teaching [9]. Although the results presented above are contextualized in China, teachers and teacher educators who hold similar teaching beliefs may find resonance here.

However, this study had some limitations. First, differences in academic reading on social media, such as reading content and reading situations, were not taken into consideration in the current study; thus, some potential influencing factors may be neglected. Second, due to the difficulty in accessing the target participants who met the requirements of this study, the sample in the quantitative research phase of this study was not large enough; thus, they could not be subdivided into different groups based on their years of teaching experience or their age. In this sense, differences in academic reading on social media caused by characteristics of the sample may not be detected. In addition, academic reading on social media is one of many situations involving teachers’ self-initiated professional learning; it is unclear whether the model established in this study can be employed to explain professional learning in other informal learning situations, such as teacher workshops or communities. These considerations still need to be explored and verified by follow-up research.

## Figures and Tables

**Table 1:** Variables, items, and sources.

Variables	Items	Sources
Perceived value of content knowledge (**PVCK**)	**PVCK1**: The content I read on social media can deepen my understanding of subject-matter knowledge.	Refs. [40,46], the results of the interviews in this study
	**PVCK2**: The content I read on social media helps me make different interpretations of subject-specific knowledge.	
	**PVCK3**: The content I read on social media can broaden my subject-matter knowledge.	
Perceived value of pedagogical content knowledge (**PVPCK**)	**PVPCK1**: The content I read on social media provides me with various teaching strategies.	Refs. [40,45]
	**PVPCK2**: The content I read on social media can help me better transfer knowledge to students.	
	**PVPCK3**: The content I read on social media enables me to better carry out activities in classroom teaching.	
Perceived value of students’ participation in the classroom (**PVSPC**)	**PVSPC1**: Using the content I read on social media in classroom teaching can lead to positive student feedback.	The results of the interviews in this study
	**PVSPC2**: Using the content I read on social media in classroom teaching can help students better understand the content I teach.	
	**PVSPC3**: Using the content I read on social media in classroom teaching can help students overcome learning difficulties.	
Intentions toward engagement with academic reading on social media (**IEARSM**)	**IEARSM1**: I will read articles and information related to my teaching on official accounts frequently.	Refs. [11,20]
	**IEARSM2**: I will follow the updates available on official accounts.	
	**IEARSM3**: I will gather information related to my teaching and research on social media.	

**Table 2:** Demographic information of participants.

Attributes	Characteristics	Frequency	%
Gender	Male	9	7%
	Female	123	93%
Age	20–30 years	13	10%
	31–40 years	84	64%
	41–50 years	25	19%
	51–60 years	9	7%
	Above 60 years of age	1	1%
Years of teaching experience	Less than 10 years	50	38%
	11–20 years	62	47%
	More than 20 years	20	15%

**Table 3:** The results for the measurement models.

	Mean (SD)	Loadings	CR	Cronbach’s α	AVE
**Criteria**		**>0.7**	**>0.7**	**>0.7**	**>0.5**
**PVCK**			0.922	0.873	0.797
PVCK1	3.568(0.939)	0.883			
PVCK2	3.462(0.965)	0.901			
PVCK3	3.864(0.860)	0.894			
**PVPCK**			0.907	0.847	0.766
PVPCK1	3.629(0.972)	0.886			
PVPCK2	3.917(0.871)	0.878			
PVPCK3	3.917(0.817)	0.862			
**PVSPC**			0.917	0.864	0.787
PVSPC1	3.735(0.815)	0.863			
PVSPC2	3.742(0.858)	0.916			
PVSPC3	3.939(0.776)	0.882			
**IEARSM**			0.842	0.719	0.641
IEARSM1	3.682(0.964)	0.808			
IEARSM2	4.235(0.920)	0.841			
IEARSM3	3.447(1.176)	0.750			

Notes. PVCK = perceived value of content knowledge, PVPCK = perceived value of pedagogical content knowledge, PVSPC = perceived value of students’ participation in the classroom, IEARSM = intentions toward engagement with academic reading on social media.

**Table 4:** Discriminant validity based on HTMT_0.90_ criteria.

	IEARSM	PVCK	PVPCK	PVSPC
**IEARSM**				
**PVCK**	0.854 [0.745, 0.951]			
**PVPCK**	0.846 [0.723, 0.953]	0.826 [0.707, 0.922]		
**PVSPC**	0.801 [0.672, 0.923]	0.733 [0.602,0.843]	0.852 [0.730, 0.951]	

Notes. PVCK = perceived value of content knowledge, PVPCK = perceived value of pedagogical content knowledge, PVSPC = perceived value of students’ participation in the classroom, IEARSM = intentions toward engagement with academic reading on social media. The results in the brackets show a 95% percentile bootstrap confidence interval with 10,000 bootstrap samples.

**Table 5:** The results for the structural model.

Hypotheses	Relationships	VIF	Path Coefficient	Standard Deviation	T Statistics	Supported	R^2^	f^2^	Q^2^
H1	PVCK ≥ IEARSM	2.191	0.358	0.079	4.502 ***	Yes	0.545	0.128	0.332
H2	PVPCK ≥ IEARSM	2.754	0.245	0.095	2.587 *	Yes		0.048	
H3	PVSPC ≥ IEARSM	2.287	0.223	0.096	2.316 *	Yes		0.048	
H4	PVCK ≥ PVPCK	1.704	0.420	0.081	5.185 ***	Yes	0.637	0.286	0.480
H5	PVSPC ≥ PVPCK	1.704	0.460	0.078	5.897 ***	Yes		0.342	

Notes. * *p* < 0.05, *** *p* < 0.001. PVCK = perceived value of content knowledge, PVPCK = perceived value of pedagogical content knowledge, PVSPC = perceived value of students’ participation in the classroom, IEARSM = intentions toward engagement with academic reading on social media.

**Table 6:** The results for the structural model.

Hypotheses	Relationships	Indirect Effects	Standard Deviation	T Statistics	Total Effects	VAF
H6	PVCK ≥ PVPCK ≥ IEARSM	0.103	0.046	2.255 *	0.461	22.34%
H7	PVSPC ≥ PVPCK ≥ IEARSM	0.112	0.051	2.222 *	0.335	33.43%

Notes. * *p* < 0.05. PVCK = perceived value of content knowledge, PVPCK = perceived value of pedagogical content knowledge, PVSPC = perceived value of students’ participation in the classroom, IEARSM = intentions toward engagement with academic reading on social media.

## Data Availability

The data of the work can be provided by the corresponding author upon request.

## Supplementary Materials

The following supporting information can be downloaded at: https://www.mdpi.com/article/10.3390/bs12100390/s1, The main items of the questionnaire.

## References

[1] Carpenter J.P., Krutka D.G. (2015). Engagement through Microblogging: Educator Professional Development via Twitter. Prof. Dev. Educ..

[2] Greenhow C., Gleason B. (2014). Social Scholarship: Reconsidering Scholarly Practices in the Age of Social Media. Br. J. Educ. Technol..

[3] Seaman J., Tinti-kane H. (2013). Social Media for Teaching and Learning.

[4] Jones W.M., Dexter S. (2014). How Teachers Learn: The Roles of Formal, Informal, and Independent Learning. Educ. Technol. Res. Dev..

[5] Lohman M.C. (2000). Environmental Inhibitors to Informal Learning in the Workplace: A Case Study of Public School Teachers. Adult Educ. Q..

[6] Liljekvist Y.E., Randahl A.C., van Bommel J., Olin-Scheller C. (2021). Facebook for Professional Development: Pedagogical Content Knowledge in the Centre of Teachers’ Online Communities. Scand. J. Educ. Res..

[7] Zhu Y. (2015). The Application of Social Media in Foreign Language Education in China (In Chinese). Technol. Enhanc. Foreign Lang. Educ..

[8] Nochumson T.C. (2020). Elementary Schoolteachers’ Use of Twitter: Exploring the Implications of Learning through Online Social Media. Prof. Dev. Educ..

[9] Borg S., Liu Y.D. (2013). Chinese College English Teachers’ Research Engagement. TESOL Q..

[10] Szeto E., Cheng A.Y.N., Hong J.C. (2016). Learning with Social Media: How Do Preservice Teachers Integrate YouTube and Social Media in Teaching?. Asia-Pacific Educ. Res..

[11] Tenopir C., Late E., Talja S., Christian L. (2019). Changes in Scholarly Reading in Finland over a Decade: Influences of E-Journals and Social Media. Sch. Inf. Sci. Fac. Publ. Other Work..

[12] Zhao W., Zong M. (2019). Relationship between WeChat Reading Quantity and CNKI Downloads for Academic Papers. Chin. J. Sci. Tech. Period..

[13] Yan C., He C. (2015). To Be or Not to Be? The “Publish or Perish” Syndrome for English Teacher Educators in China. Front. Educ. China.

[14] Lee I. (2014). Publish or Perish: The Myth and Reality of Academic Publishing. Lang. Teach..

[15] Yuan R. (2017). ‘This Game Is Not Easy to Play’: A Narrative Inquiry into a Novice EFL Teacher Educator’s Research and Publishing Experiences. Prof. Dev. Educ..

[16] Hu P., Chen J. (2014). A Qualitative Study on Academic Reading of College English Teachers: Beliefs towards Academic Reading, Reading Strategies and Reading Difficulties. Foreign Lang. World.

[17] Rankin J., Becker F. (2006). Does Reading the Research Make a Difference? A Case Study of Teacher Growth in FL German. Mod. Lang. J..

[18] Kirkwood M., Christie D. (2006). The Role of Teacher Research in Continuing Professional Development. Br. J. Educ. Stud..

[19] Hedgcock J.S. (2002). Toward a Socioliterate Approach to Second Language Teacher Education. Mod. Lang. J..

[20] Tenopir C., Volentine R., King D.W. (2013). Social Media and Scholarly Reading. Online Inf. Rev..

[21] Ross L., Sennyey P. (2008). The Library Is Dead, Long Live the Library! The Practice of Academic Librarianship and the Digital Revolution. J. Acad. Librariansh..

[22] Deodato J. (2018). Overhyped Fad or Missed Opportunity? A History of Academic Libraries and the Social Web. J. Web Librariansh..

[23] Xu J., Kang Q., Song Z., Clarke C.P. (2015). Applications of Mobile Social Media: WeChat Among Academic Libraries in China. J. Acad. Librariansh..

[24] Holmberg K., Thelwall M. (2014). Disciplinary Differences in Twitter Scholarly Communication. Scientometrics.

[25] Ohata K., Fukao A. (2014). L2 Learners’ Conceptions of Academic Reading and Themselves as Academic Readers. System.

[26] Greenhow C., Lewin C. (2016). Social Media and Education: Reconceptualizing the Boundaries of Formal and Informal Learning. Learn. Media Technol..

[27] Latchem C. (2018). Open and Distance Non-Formal Education in Developing Countries.

[28] Boyd D.M., Ellison N.B. (2007). Social Network Sites: Definition, History, and Scholarship. J. Comput. Commun..

[29] Mei B., Brown G.T.L., Teo T. (2018). Toward an Understanding of Preservice English as a Foreign Language Teachers’ Acceptance of Computer-Assisted Language Learning 2.0 in the People’s Republic of China. J. Educ. Comput. Res..

[30] Teo T., Sang G., Mei B., Hoi C.K.W. (2019). Investigating Pre-Service Teachers’ Acceptance of Web 2.0 Technologies in Their Future Teaching: A Chinese Perspective. Interact. Learn. Environ..

[31] Davis F.D. (1989). Perceived Usefulness, Perceived Ease of Use, and User Acceptance of Information Technology. MIS Q..

[32] Al-Rahmi W.M., Alias N., Othman M.S., Marin V.I., Tur G. (2018). A Model of Factors Affecting Learning Performance through the Use of Social Media in Malaysian Higher Education. Comput. Educ..

[33] Alamri M.M., Almaiah M.A., Al-Rahmi W.M. (2020). Social Media Applications Affecting Students’ Academic Performance: A Model Developed for Sustainability in Higher Education. Sustainability.

[34] Sharma S.K., Joshi A., Sharma H. (2016). A Multi-Analytical Approach to Predict the Facebook Usage in Higher Education. Comput. Hum. Behav..

[35] Amadu L., Muhammad S.S., Mohammed A.S., Owusu G., Lukman S. (2018). Using Technology Acceptance Model to Measure the Ese of Social Media for Collaborative Learning in Ghana. J. Technol. Sci. Educ..

[36] Rauniar R., Rawski G., Yang J., Johnson B. (2014). Technology Acceptance Model (TAM) and Social Media Usage: An Empirical Study on Facebook. J. Enterp. Inf. Manag..

[37] Venkatesh V., Morris M.G., Davis G.B., Davis F.D. (2003). User Acceptance of Information Technology: Toward a Unified View. MIS Q..

[38] Gruzd A., Staves K., Wilk A. (2012). Connected Scholars: Examining the Role of Social Media in Research Practices of Faculty Using the UTAUT Model. Comput. Hum. Behav..

[39] Dermentzi E., Papagiannidis S. (2018). UK Public’s Intention to Engage with Academia via Online Technologies. Behav. Inf. Technol..

[40] Hwang M.Y., Hong J.C., Hao Y.W. (2018). The Value of CK, PK, and PCK in Professional Development Programs Predicted by the Progressive Beliefs of Elementary School Teachers. Eur. J. Teach. Educ..

[41] Shulman L.S. (1987). Knowledge and Teaching: Foundations of the New Reform. Harv. Educ. Rev..

[42] Gess-Newsome J., Taylor J.A., Carlson J., Gardner A.L., Wilson C.D., Stuhlsatz M.A.M. (2017). Teacher Pedagogical Content Knowledge, Practice, and Student Achievement. Int. J. Sci. Educ..

[43] Olfos R., Goldrine T., Estrella S. (2014). Teachers’ Pedagogical Content Knowledge and Its Relation with Students’ Understanding. Rev. Bras. Educ..

[44] Park S., Chen Y.C. (2012). Mapping out the Integration of the Components of Pedagogical Content Knowledge (PCK): Examples from High School Biology Classrooms. J. Res. Sci. Teach..

[45] Park S., Oliver J.S. (2008). Revisiting the Conceptualisation of Pedagogical Content Knowledge (PCK): PCK as a Conceptual Tool to Understand Teachers as Professionals. Res. Sci. Educ..

[46] Baumert J., Kunter M., Blum W., Brunner M., Voss T., Jordan A., Klusmann U., Krauss S., Neubrand M., Tsai Y.M. (2010). Teachers’ Mathematical Knowledge, Cognitive Activation in the Classroom, and Student Progress. Am. Educ. Res. J..

[47] Kleickmann T., Richter D., Kunter M., Elsner J., Besser M., Krauss S., Baumert J. (2013). Teachers’ Content Knowledge and Pedagogical Content Knowledge: The Role of Structural Differences in Teacher Education. J. Teach. Educ..

[48] Friedrichsen P.J., Abell S.K., Pareja E.M., Brown P.L., Lankford D.M., Volkmann M.J. (2009). Does Teaching Experience Matter? Examining Biology Teachers’ Prior Knowledge for Teaching in an Alternative Certification Program. J. Res. Sci. Teach..

[49] Sorge S., Keller M.M., Neumann K., Möller J. (2019). Investigating the Relationship between Pre-Service Physics Teachers’ Professional Knowledge, Self-Concept, and Interest. J. Res. Sci. Teach..

[50] Merk S., Rosman T., Rueß J., Syring M., Schneider J. (2017). Pre-Service Teachers’ Perceived Value of General Pedagogical Knowledge for Practice: Relations with Epistemic Beliefs and Source Beliefs. PLoS ONE.

[51] Ajjan H., Hartshorne R. (2008). Investigating Faculty Decisions to Adopt Web 2.0 Technologies: Theory and Empirical Tests. Internet High. Educ..

[52] Dermentzi E., Papagiannidis S., Osorio Toro C., Yannopoulou N. (2016). Academic Engagement: Differences between Intention to Adopt Social Networking Sites and Other Online Technologies. Comput. Human Behav..

[53] Manca S., Ranieri M. (2016). Facebook and the Others. Potentials and Obstacles of Social Media for Teaching in Higher Education. Comput. Educ..

[54] Creswell W.J., Creswell J.D. (2018). Research Design: Qualitative, Quantitative and Mixed Methods Approaches.

[55] Francis J.J., Johnston M., Clare R., Liz G., Entwistle V., Eccles M.P., Grimshaw J.M. (2010). What Is an Adequate Sample Size? Operationalising Data Saturation for Theory-Based Interview Studies. Psychol. Health.

[56] Hesse-Biber S. (2018). Gender Differences in Psychosocial and Medical Outcomes Stemming From Testing Positive for the BRCA1/2 Genetic Mutation for Breast Cancer: An Explanatory Sequential Mixed Methods Study. J. Mix. Methods Res..

[57] Yan J.X., Horwitz E.K. (2008). Learners’ Perceptions of How Anxiety Interacts with Personal and Instructional Factors to Influence Their Achievement in English: A Qualitative Analysis of EFL Learners in China. Lang. Learn..

[58] Underwood P.R. (2012). Teacher Beliefs and Intentions Regarding the Instruction of English Grammar under National Curriculum Reforms: A Theory of Planned Behaviour Perspective. Teach. Teach. Educ..

[59] Hair J., Hult G.T., Ringle C., Sarstedt M. (2017). A Primer on Partial Least Squares Structural Equation Modeling (PLS-SEM).

[60] Kock N. (2015). Common Method Bias in PLS-SEM. Int. J. e-Collaboration.

[61] Hair J.F., Hult G.T.M., Ringle C.M., Sarstedt M. (2014). A Primer on Partial Least Squares Structural Equation Modeling (PLS-SEM).

[62] Hu L., Bentler P.M. (1998). Fit Indices in Covariance Structure Modeling: Sensitivity to Underparameterized Model Misspecification. Psychol. Methods.

[63] Zhao X., Lynch J.G., Chen Q. (2010). Reconsidering Baron and Kenny: Myths and Truths about Mediation Analysis. J. Consum. Res..

[64] Borg M. (2003). Teachers’ Beliefs. ELT J..

[65] Kagan D.M. (1992). Implication of Research on Teacher Belief. Educ. Psychol..

[66] De Vries S., Jansen E.P.W.A., van de Grift W.J.C.M. (2013). Profiling Teachers’ Continuing Professional Development and the Relation with Their Beliefs about Learning and Teaching. Teach. Teach. Educ..

[67] Shulman L.S. (1986). Those Who Understand: Knowledge Growth in Teaching. Educ. Res..

[68] Borg S. (2010). Language Teacher Research Engagement. Lang. Teach..

[69] Yan C., He C., Guo X., Wang J. (2020). Plateauing of Chinese Female Mid-Career EFL Teacher Educators at Regional Teacher Education Universities. Prof. Dev. Educ..

[70] Han J., Zhao Y., Liu M., Zhang J. (2021). The Development of College English Teachers’ Pedagogical Content Knowledge (PCK): From General English to English for Academic Purposes. Asia Pacific Educ. Rev..

[71] Sugimoto C.R., Work S., Larivière V., Haustein S. (2017). Scholarly Use of Social Media and Altmetrics: A Review of the Literature. J. Assoc. Inf. Sci. Technol..

