# Supplementary material for: Chinese University English Teachers’ Professional Learning through Academic Reading on Social Media—A Mixed-Methods Approach
Source: Behav Sci (Basel). 2022 Oct 12;12(10):390. doi: 10.3390/bs12100390 (PMC9598396; doi:10.3390/bs12100390)
Supplement: Supplementary file 1 [file behavsci-12-00390-s001.zip › behavsci-1946032-supplementary.pdf]

Supplementary Material

The main items of the questionnaire of *Chinese university English teachers' professional learning through academic reading on social media- A mixed-methods approach*

Section 1: The experience of using social media for academic purposes

1. Have you ever read academic resources or information on social media (e.g., WeChat, Weibo, Facebook, and Twitter)? (If you do not have the experience of reading academic resources or information on social media, you can stop answering this questionnaire.)
□Yes
□No

2. On which social media do you usually read academic resources or information? (Choose one or more than one option)
□ WeChat
□ Weibo
□ Facebook
□ Twitter
□ Zhihu
□ Other ________

3. In the past month, how much time (in hours) did you spend reading academic resources and information on social media every day? ________

Section 2. Behavioral intentions and factors influencing intentions toward engagement with academic reading on social media

Perceived value of content knowledge (PVCK)
4. The content I read on social media can deepen my understanding of the subject-matter knowledge.

□	□	□	□	□	□	□
1	2	3	4	5	6	7

5. The content I read on social media helps me make different interpretations of subject-specific knowledge.

□	□	□	□	□	□	□
1	2	3	4	5	6	7

6. The content I read on social media can broaden my subject-matter knowledge.

□	□	□	□	□	□	□
1	2	3	4	5	6	7

Perceived value of pedagogical content knowledge (PVPCK)

7. The content I read on social media provides me with various teaching strategies.

☐	☐	☐	☐	☐	☐	☐
1	2	3	4	5	6	7

8. The content I read on social media can help me better transfer knowledge to students.

☐	☐	☐	☐	☐	☐	☐
1	2	3	4	5	6	7

9. The content I read on social media enables me to better carry out activities in classroom teaching.

☐	☐	☐	☐	☐	☐	☐
1	2	3	4	5	6	7

Perceived value of students' participation in the classroom (PVSPC)

10. Using the content I read on social media in classroom teaching can lead to positive student feedback.

☐	☐	☐	☐	☐	☐	☐
1	2	3	4	5	6	7

11. Using the content I read on social media in classroom teaching can help students better understand the content I teach.

☐	☐	☐	☐	☐	☐	☐
1	2	3	4	5	6	7

12. Using the content I read on social media in classroom teaching can help students overcome learning difficulties.

☐	☐	☐	☐	☐	☐	☐
1	2	3	4	5	6	7

Intentions toward engagement with academic reading on social media (IEARSM)

13. I will read articles and information related to my teaching on official accounts frequently.

☐	☐	☐	☐	☐	☐	☐
1	2	3	4	5	6	7

14. I will follow the updates available on official accounts.

☐	☐	☐	☐	☐	☐	☐
1	2	3	4	5	6	7

15. I will gather information related to my teaching and research on social media.

☐	☐	☐	☐	☐	☐	☐
1	2	3	4	5	6	7

Section 3. Demographics

Gender: ____

Your age: ____

Your years of teaching experience as a university English teacher: ____

Your university where currently you work in: ____
